# Investigation of a COVID-19 outbreak on the Charles de Gaulle aircraft carrier, March to April 2020: a retrospective cohort study

**DOI:** 10.2807/1560-7917.ES.2022.27.21.2100612

**Published:** 2022-05-26

**Authors:** Franck de Laval, Hervé Chaudet, Olivier Gorgé, Joffrey Marchi, Constance Lacrosse, Aissata Dia, Vanessa Marbac, Bakridine Mmadi Mrenda, Gaëtan Texier, Flavie Letois, Charles Chapus, Véronique Sarilar, Jean-Nicolas Tournier, Anthony Levasseur, Jacques Cobola, Flora Nolent, Fabien Dutasta, Frédéric Janvier, PA-CDG COVID-19 investigation group, Jean-Baptiste Meynard, Vincent Pommier de Santi, PA-CDG COVID-19 investigation group, Marion Fossier, Christelle Tong, Liliane Pellegrin, Sandrine Duron, Marie Dubois, Lénaïck Ollivier, Catherine Désideri Vaillant, Brice Schneider, Olivier Ferraris, Fabrice Biot, Noémie Verguet, Frédéric Iseni, Gaëlle Frenois-Veyrat, Marine Ridet, Marc Grandadam, Laurent Martinez, Jean-François Louis, Fabienne Lafrogne, Eric Reverbel, Jean-Marie Loreau, Yann Morin, Albane de Bonet d’Oléon, Lise Holterbach, Audrey Merens, Vincent Foissaud, Léopoldine Peron, Sophie Murris, Nastasia Menoud, Anne-Claire Garcia, Typhaine Ressort

**Affiliations:** 1 French Armed Forces Center for Epidemiology and Public Health (CESPA), Marseille, France; 2 Aix-Marseille University, INSERM, IRD, SESSTIM (Economic and Social Sciences, Health Systems, and Medical Informatics), Marseille, France; 3 Aix-Marseille University, IRD, AP-HM, SSA (French Military Health Service), VITROME, Marseille, France; 4 University Hospital Institute Méditerranée Infection, Marseille, France; 5 French Armed Forces Biomedical Research Institute (IRBA), Brétigny-sur-Orge, France; 6 French Military Health Service, Toulon, France; 7 Aix Marseille University, IRD, AP-HM, MEPHI, Marseille, France; 8 Sainte-Anne Military Teaching Hospital, Toulon, France; 9 PA-CDG COVID-19 investigation group members are listed under Collaborators.

**Keywords:** SARS-CoV-2, COVID-19, Outbreak, Aircraft Carrier, Ship, Herd immunity

## Abstract

**Background:**

SARS-CoV-2 emergence was a threat for armed forces. A COVID-19 outbreak occurred on the French aircraft carrier Charles de Gaulle from mid-March to mid-April 2020.

**Aim:**

To understand how the virus was introduced, circulated then stopped circulation, risk factors for infection and severity, and effectiveness of preventive measures.

**Methods:**

We considered the entire crew as a cohort and collected personal, clinical, biological, and epidemiological data. We performed viral genome sequencing and searched for SARS-CoV-2 in the environment.

**Results:**

The attack rate was 65% (1,148/1,767); 1,568 (89%) were included. The male:female ratio was 6.9, and median age was 29 years (IQR: 24–36). We examined four clinical profiles: asymptomatic (13.0%), non-specific symptomatic (8.1%), specific symptomatic (76.3%), and severe (i.e. requiring oxygen therapy, 2.6%). Active smoking was not associated with severe COVID-19; age and obesity were risk factors. The instantaneous reproduction rate (R_t_) and viral sequencing suggested several introductions of the virus with 4 of 5 introduced strains from within France, with an acceleration of R_t_ when lifting preventive measures. Physical distancing prevented infection (adjusted OR: 0.55; 95% CI: 0.40–0.76). Transmission may have stopped when the proportion of infected personnel was large enough to prevent circulation (65%; 95% CI: 62–68).

**Conclusion:**

Non-specific clinical pictures of COVID-19 delayed detection of the outbreak. The lack of an isolation ward made it difficult to manage transmission; the outbreak spread until a protective threshold was reached. Physical distancing was effective when applied. Early surveillance with adapted prevention measures should prevent such an outbreak.

## Introduction

Infectious diseases are a common threat in the armed forces [1], which can experience epidemics that impact their capabilities or, alternatively, personnel may introduce pathogens into naive populations [2]. Controlling infectious diseases in the armed forces is a challenge, and military health services must be able to detect, investigate, and react to the emergence of new pathogens and outbreaks [3]. For example, a military ship’s crew is a cohort-like population, with members living closely together in a confined space; such environments can be catalysts for virus transmissions. The emergence and spread of the severe acute respiratory syndrome coronavirus 2 (SARS-CoV-2) virus [4] and the related disease coronavirus disease (COVID-19) across Europe in early 2020 exemplify such a threat, as evidenced by COVID-19 outbreaks on the Diamond Princess cruise ship [5] and the USS Theodore Roosevelt aircraft carrier [6]. At that time, this virus, its transmission, and the symptoms of the disease were unknown [7,8].

In January 2020, the French Carrier Battle Group (CVBG), including the aircraft carrier Charles de Gaulle (PA-CDG) and its carrier air wing, left the city of Toulon, its home port in South-eastern France, for a 4-month mission. From February to April, the CVBG navigated between the Mediterranean and the North Sea, making only two stopovers in Limassol (Cyprus, 21–26 February) and Brest (Western France, 13–16 March). In addition, the PA-CDG, as a hub airport at sea, took many service members on board, directly from land or from the other ships of the CVBG. On 7 April 2020, a COVID-19 outbreak was reported on the Charles de Gaulle. An investigation team was sent on board to perform testing and confirm the diagnosis. Exhaustive case identification and systematic isolation was performed once the PA-CDG docked in Toulon on 13 April, and the crew disembarked.

Our aim was to describe the entire outbreak, including a hypothesis to explain how the virus was introduced on board. In addition, we present the clinical profile of the COVID-19 cases including risk factors for infection and severity, the effectiveness of preventive measures and the potential protective threshold in our setting.

## Methods

### Study design

The investigation was conducted as a retrospective cohort by the French Armed Forces Center for Epidemiology and Public Health (CESPA) among the 1,767 service members of the PA-CDG present on board between 21 February and 13 April 2020.

Our study started with the stopover in Limassol, as there was no SARS-CoV-2 circulation identified in France and Europe when the PA-CDG departed Toulon on 21 January 2020 [4]. The complete timeline of the PA-CDG journey is available in Supplementary Figure S1.

### Data sources

From 23 to 30 April, each service member was phoned by investigators with experience conducting a retrospective survey, using a standardised questionnaire. We chose this type of investigation because service members were isolated or quarantined. The questions were about personal (sex, age, body mass index (BMI), active smoking, blood group and rhesus factor), clinical (medical history, presence of symptoms, and dates of symptom onset/disappearance), and epidemiological data (date of boarding, places where they slept/had meals/worked, possible exposure to COVID-19, 1-to-3 score (never/sometimes, often and always) for application of physical distancing > 1 m, face mask wearing, and hand hygiene). When necessary, we obtained additional information from medical records and consultation registers. The first 30 confirmed COVID-19 cases were more extensively interviewed for precise dates and close contacts and/or possible exposure to other COVID-19 cases during the 14 days before symptom onset.

### Case definitions

A biologically confirmed COVID-19 case was any service member with a positive SARS-CoV-2 quantitative (q)RT-PCR test. A confirmed COVID-19 case was any service member with a positive SARS-CoV-2 qRT-PCR and/or presenting symptoms of anosmia and/or ageusia. We included anosmia and ageusia in the definition because of their high specificity for COVID-19 disease, in order to identify cases earlier in those whose qRT-PCR test was negative at the time of investigation [9].

For this investigation, we used the following four clinical profile definitions: (i) asymptomatic case (no symptoms within 14 days of a positive SARS-CoV-2 qRT-PCR test), (ii) specific symptomatic (with symptoms indicative of COVID-19, e.g. anosmia, ageusia, cough, or fever), (iii) non-specific symptomatic (with other symptoms), and (iv) severe (requiring oxygen therapy).

### Biological confirmation and testing strategy

A first panel of 67 patients presenting ongoing symptoms was sampled on board on 8 April 2020, confirming SARS-CoV-2 as the pathogen responsible for the outbreak. Then, from 14 to 17 April, all the service members underwent clinical screening and testing after they disembarked. Sampling was performed under biosecurity conditions using nasopharyngeal swabs. The qRT-PCR tests were analysed using either the LightCycler480 System (Roche Diagnostics) with assays set-up by the French Armed Forces Biomedical Research Institute (IRBA, Brétigny-sur-Orge) for samples taken on 8 April, and for later samples the cobas 6800 system with cobas SARS-CoV-2 kits (Roche Diagnostics) to target the Orf1a/b domain, performed according to the manufacturer’s instructions. Results were qualitative and the Cq threshold for a positive test was 35. Biologically confirmed cases were isolated at a military compound or a hospital in the Toulon area depending on the severity of their case, and those who tested negative were quarantined for 14 days. They were monitored daily by medical staff and tested if symptoms arose. All the qRT-PCR results were collected and used for the investigation. A negative qRT-PCR was required to leave the quarantine.

SARS-CoV-2 qRT-PCR was also performed on environmental swabs sampled from several common objects or surfaces before disinfection and inside the air conditioning system of the aircraft carrier.

### Viral genome sequencing

Viral genome sequencing was performed on the first 60 positive samples taken from the service members on 8 April to contribute to molecular epidemiological studies (method in Supplementary Material: viral genome sequencing). Viral sequences are available online in the GISAID database (https://www.gisaid.org). The anonymous individual identifiers, GISAID accession numbers, and other data can be found in the Supplementary Table S1.

### Statistical analysis

All statistical analyses were done using R [10]. We first studied the outbreak dynamics based on the epidemic curves and the instantaneous reproduction rate (R_t_; Supplementary Methods: study of instantaneous reproduction rate). Then, we studied the circulation of the virus between sleeping compartments and work areas; the results are summarised in a chord diagram. Finally, we identified risk factors for SARS-CoV-2 infection and severity of COVID-19 using stepwise logistic regression, a descending step-by-step method that considers variables with p < 0.25 in univariate analysis or having an effect already recognised in other studies (especially age). We also tested a random-effect model on sleeping compartments, mess halls, and work areas; it was only significant for work areas.

## Results

### Population

**Figure 1:**
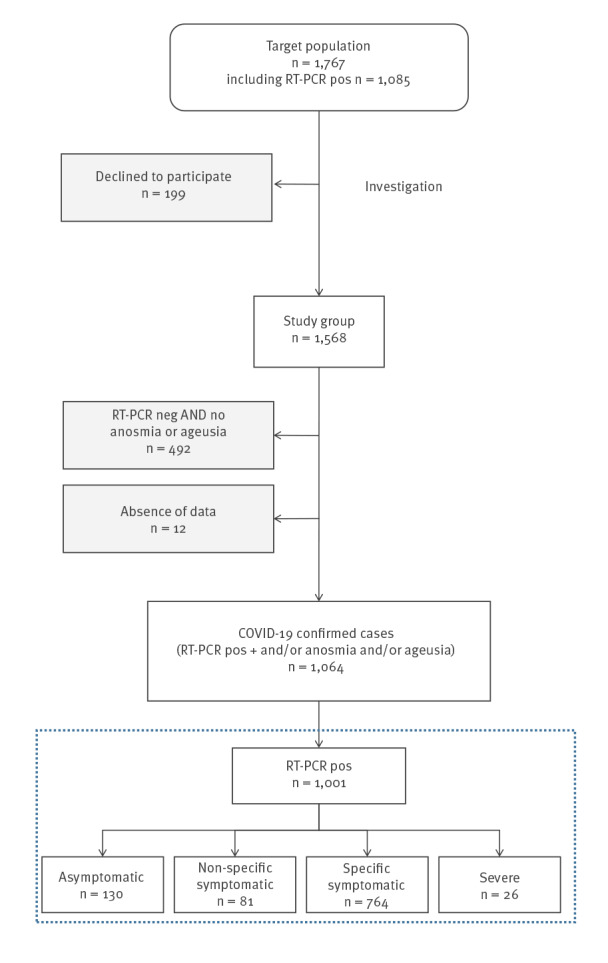
Flowchart of the COVID-19 outbreak investigation on the Charles de Gaulle aircraft carrier, April 2020

COVID-19: coronavirus disease; neg: negative; pos; positive.

Of 1,767 service members on the PA-CDG, the overall attack rate for COVID-19-confirmed cases was 65.0% (1,148/1,767). Among these, 1,568 (88.7%) were included in the study (Figure 1) to describe the outbreak, and 199 (11.3%) did not participate in the outbreak description (177 did not answer their mobile phone despite several calls and information messages, and 22 declined the offer to participate). Both participating vs non-participating groups differed in their mean ages (31.0 vs 27.8, p < 0.001), and in their RT-PCR positivity rates (64.7% vs 43.8, p < 0.001).

See details of data inside the blue dashed line in Table 1.

In the study group (n = 1,568), the sex ratio was 6.9 (1,369 men/199 women), and median age was 29 years (interquartile range (IQR): 24–36). Median body mass index (BMI) was 24.2 (IQR: 22.2–26.1). Several service members (n = 21; 1.3%) presented risk factors for severe COVID-19: arterial hypertension (n = 14), respiratory disease (n = 6), and diabetes (n = 1). Of the 1,568, 568 (36.2%) were active smokers. There were 593 (38.3%) crew members, 773 (49.7%) non-commissioned officers, and 187 (12.0%) officers. In the study group, 1,064 (67.9%) were COVID-19-confirmed cases, including 1,001 (94.1%) biologically confirmed cases and 63 (5.9%) cases with an early onset of anosmia and/or ageusia and a negative qRT-PCR at the time of investigation.

### Clinical presentation, risk factors and exposure of COVID-19 cases

Among the 1,001 biologically confirmed cases, we describe four clinical profiles: asymptomatic (n = 130; 13.0%), non-specific symptomatic (n = 81; 8.1%), specific symptomatic (n = 764; 76.3%), and severe (n = 26; 2.6%) (Table 1). Median duration of symptoms at the time of the investigation was 8 days (IQR: 4–12). Age (p < 0.001) and BMI (p < 0.02) increased with the intensity of the clinical profile, in contrast to active smoking, which decreased (p = 0.003).

**Table 1:** Demographic characteristics, risk factors, and symptoms according to clinical profile among biologically confirmed COVID-19 cases, Charles de Gaulle aircraft carrier, April 2020 (n = 1,001)

Variables	Total positive RT-PCR n = 1,001^a^		Clinical profile								p value for clinical profile^b^
			Asymptomatic n = 130^a^		Non-specific symptomatic n = 81^a^		Specific symptomatic n = 764^a^		Severe n = 26^a^		
	n	%	n	%	n	%	n	%	n	%	
Sex											
Female	121	12.1	12	9.2	11	13.6	95	12.4	3	11.5	0.74
Male	880	87.9	118	90.8	70	86.4	669	87.6	23	88.5	
Age (years)											
18–25	314	31.4	51	39.2	30	37.0	231	30.3	2	7.7	< 0.001
26–35	414	41.4	56	43.1	34	42.0	321	42.1	3	11.5	
36–45	182	18.2	20	15.4	13	16.0	139	18.2	10	38.5	
46–60	90	9.0	3	2.3	4	4.9	72	9.4	11	42.3	
Hypertension											
No	985	98.4	129	100.0	80	100.0	751	98.8	25	96.2	0.21
Yes	10	1.0	0	0.0	0	0.0	9	1.2	1	3.8	
Respiratory illness											
No	990	98.9	129	100.0	80	100.0	755	99.3	26	100.0	0.67
Yes	5	0.5	0	0.0	0	0.0	5	0.7	0	0.0	
Active smoking											
No	676	67.5	78	60.0	51	63.0	523	68.5	24	96.0	0.003
Yes	323	32.3	52	40.0	30	37.0	240	31.5	1	4.0	
BMI ≥ 25											
No	597	59.6	80	61.5	47	58.8	462	61.4	8	30.8	0.02
Yes	391	39.1	50	38.5	33	41.2	290	38.6	18	69.2	
Symptoms											
Fever	449	44.9	NA	NA	NA	NA	425	55.6	24	92.3	NA
Cough	356	35.6			NA	NA	333	43.6	23	88.5	
Headache	562	56.1			45	55.6	497	65.1	20	76.9	
Asthenia	459	45.9			30	37.0	411	53.8	18	69.2	
Anosmia	550	54.9			NA	NA	539	70.5	11	42.3	
Ageusia	446	44.6			NA	NA	436	57.1	10	38.5	
Rhinitis	379	37.9			31	38.3	340	44.5	8	30.8	
Myalgia	448	44.8			24	29.6	400	52.4	24	92.3	
Odynophagia	155	15.5			10	12.3	142	18.6	3	11.5	
Earache	35	3.5			0	NA	34	4.5	1	3.8	
Malaise	31	3.1			0	NA	28	3.7	3	11.5	
Dyspnoea	272	27.2			6	7.4	247	32.3	19	73.1	
Chest pain	131	13.1			8	9.9	111	14.5	12	46.2	
Diarrhoea	150	15.0			8	9.9	128	16.8	14	53.8	
Vomiting	24	2.4			3	3.7	18	2.4	3	11.5	
Abdominal pain	61	6.1			1	1.2	55	7.2	5	19.2	
Other	61	6.1			4	4.9	54	7.1	3	11.5	

BMI: body mass index; COVID-19: coronavirus disease; NA: not applicable.

^a^ Missing data explain the differences between totals of each variable.

^b^ Chi-squared test.

We examined risk factors for infection in the entire study group but excluding cases with missing data for the model (Table 2). Active smoking was associated with reduced infection (adjusted odds ratio (OR_a_): 0.57; 95% confidence interval (CI): 0.44–0.73), contrary to age group (46–60 vs 18–25 years) (OR_a_: 1.79; 95% CI: 1.03–3.20). Sex, blood group, BMI and a history of risk factors were not associated with SARS-CoV-2 infection.

**Table 2:** Logistic regression analysis of possible factors associated with COVID-19-confirmed cases, Charles de Gaulle aircraft carrier, April 2020 (n = 1,221^a^)

Variables	Univariate analysis			Multivariate analysis		
	OR	95% CI	p value	OR_a_	95% CI	p value
**Individual characteristics**						
Sex						
Female	Ref			NA		
Male	1.28	0.91–1.79	0.15			
Age (years)^b^						
18–25	Ref					
26–35	1.17	0.88–1.55	0.29	1.30	0.95–1.78	0.10
36–45	0.95	0.67–1.34	0.75	1.05	0.73–1.51	0.81
46–60	1.64	0.98–2.84	0.07	1.79	1.03–3.20	0.04
Active smoking						
No	Ref					
Yes	0.55	0.43–0.71	< 0.001	0.57	0.44–0.73	< 0.001
BMI ≥ 25						
No	Ref			NA		
Yes	1.12	0.88–1.44	0.36			
Blood group						
A	Ref			NA		
AB	1.30	0.68–2.67	0.45			
B	0.78	0.52–1.18	0.23			
O	0.87	0.67–1.13	0.30			
Rhesus factor						
Negative	Ref			NA		
Positive	0.90	0.63–1.29	0.58			
**Infection prevention measures**						
Physical distancing						
Never/sometimes	Ref					
Often	0.85	0.64–1.14	0.27	0.91	0.68–1.23	0.54
Always	0.51	0.37–0.69	< 0.001	0.55	0.40–0.76	< 0.001
Hand hygiene						
Never/sometimes	Ref			NA		
Often	0.91	0.38–1.99	0.82			
Always	0.75	0.33–1.58	0.47			
**Environment**						
Mess hall						
Crew	Ref					
Non-commissioned officer	1.10	0.85–1.42	0.47	1.01	0.76–1.34	0.93
Officer	0.76	0.50–1.17	0.21	0.61	0.38–0.98	0.04
Work Area						
Flight deck	4.03	2.10–8.72	< 0.001	4.09	2.10–8.97	< 0.001
Other	Ref					
Sleeping compartment occupancy						
1–4	Ref			NA		
5–10	1.03	0.73–1.46	0.86			
11–15	1.10	0.80–1.52	0.56			
> 15	1.12	0.77–1.63	0.55			

BMI: body mass index; CI: confidence interval; COVID-19: coronavirus disease; NA: not applicable; OR: odds ratio; OR_a_: adjusted odds ratio; Ref.: reference.

^a^ Study group (n = 1,568) excluding cases with missing data for the model (n = 347).

^b^ Age was forced into the model for the multivariate analysis.

#### Preventive measures

We evaluated the effectiveness of infection prevention measures. Application of physical distancing was associated with SARS-CoV-2 infection (OR_a_: 0.55; 95% CI: 0.40–0.76), contrary to the score of hand hygiene. Since face masks were required only for cases with symptoms indicative of COVID-19 on board in March, and for all service members once the epidemic was confirmed on 8 April, this prevention measure could not be studied in statistical analyses.

We also examined the infection risk in sleeping, eating, and working areas. Working on the flight deck led to greater odds of SARS-CoV-2 infection (OR_a_: 4.09; 95% CI: 2.10–8.97) than the other areas; it was where the outbreak began. The officers’ mess hall (which is like a restaurant where personnel density was low during meals) was associated with lower odds (OR_a_: 0.61; 95% CI: 0.38–0.98) than the mess hall used by the crew (self-service and high density).

#### Disease severity

A total of 29 severe cases were recorded. Of these, 26 had a positive qRT-PCR (details are in Table 1). Three cases required hospitalisation in an intensive care unit, none died. We examined risk factors for severity among confirmed COVID-19 cases: Active smoking was inversely associated with severe COVID-19 (OR_a_: 0.12; 95% CI: 0.01–0.57). Conversely, belonging to the oldest age group (OR_a_: 8.81; 95% CI: 1.01–61.08) and having a BMI above 25 (OR_a_: 3.12; 95% CI: 1.22–9.02) were risk factors for severity (Table 3).

**Table 3:** Logistic regression analysis of exposure factors linked to severity of COVID-19, Charles de Gaulle aircraft carrier, April 2020 (n = 845^a^)

Variables	Univariate analysis			Multivariate analysis		
	OR	95% CI	p value	OR_a_	95% CI	p value
Sex						
Female	Ref			NA		
Male	0.88	0.29–3.79	0.83			
Age (years)						
18–25	Ref					
26–35	1.47	1.28–10.65	0.66	1.19	0.23–8.70	0.84
36–45	7.07	1.75–47.29	0.01	4.76	1.14–32.30	0.05
46–60	14.4	1.40–98.54	0.001	8.81	1.01–61.08	0.008
Active smoking						
No	Ref					
Yes	0.09	0.01–0.45	0.02	0.12	0.01–0.57	0.04
BMI ≥ 25						
No	Ref					
Yes	3.92	1.57–11.08	0.005	3.12	1.22–9.02	0.02
Blood group						
A	Ref			NA		
AB	1.16	0.06–6.59	0.89			
B	1.01	0.15–4.13	0.99			
O	1.20	0.47–3.18	0.70			
Rhesus factor						
Negative	Ref			NA		
Positive	0.98	0.33–4.24	0.98			

BMI: body mass index; CI: confidence interval; COVID-19: coronavirus disease; NA: not applicable; OR: odds ratio; OR_a_: adjusted OR.

^a^ COVID-19-confirmed cases (n = 1,001) in the study group, excluding cases with missing data for the model (n = 156).

### Epidemiological description of the outbreak

**Figure 2:**
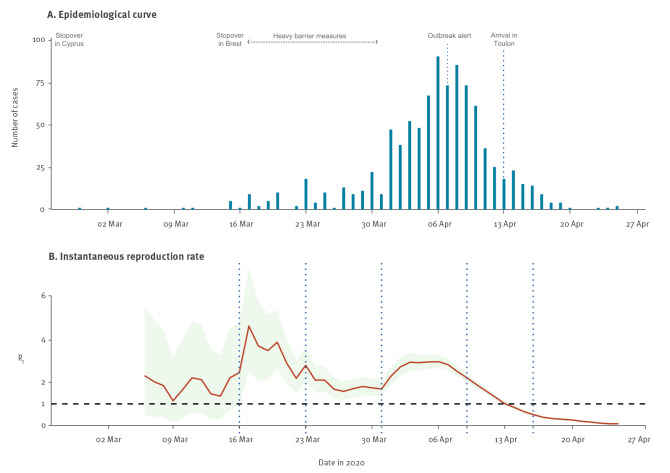
A. Epidemiological curve of confirmed COVID-19 cases according to date of symptom onset and B. Instantaneous reproduction rate curve of the Charles de Gaulle aircraft carrier, April 2020 (n = 959^a^)

Among all 1,064 confirmed COVID-19 cases, a date of symptom onset was available for 959 (90.1%), from which an epidemiological curve associated with the progression of estimated R_t_ (Figure 2) was generated. Final R during the outbreak was 2.86. Of all service members on board, the final proportion of COVID-19 confirmed cases was 65.0% (1,148/1,767; 95% CI: 62–68).

COVID-19: coronavirus disease; R_t_: instantaneous reproduction rate.

^a^ COVID-19 confirmed cases in the study group and with a date of symptoms onset

In panel B, the red line indicates the estimated R_t_ progression. The green shaded area represents the 95% confidence interval. The blues dotted lines delimit five R_t_ main changes.

The statistically significant changes in the epidemiological and R_t_ curves highlighted five phases in the progression of R_t_: (i) an initial phase of low circulation with sporadic cases (1.1 < R_t_ < 2.2), (ii) an acceleration following the Brest stopover associated with R_t_ peaking at 3.9, (iii) a phase of moderate virus circulation starting between 21 and 24 March, with R_t_ between 1 and 2, (iv) an epidemic phase starting between 31 March and 1 April, with R_t_ peaking at 2.9, and (v) a slowing down of the epidemic, which began on 8 April and crossed the level of R_t_ = 1 on 13 April.

**Figure 3:**
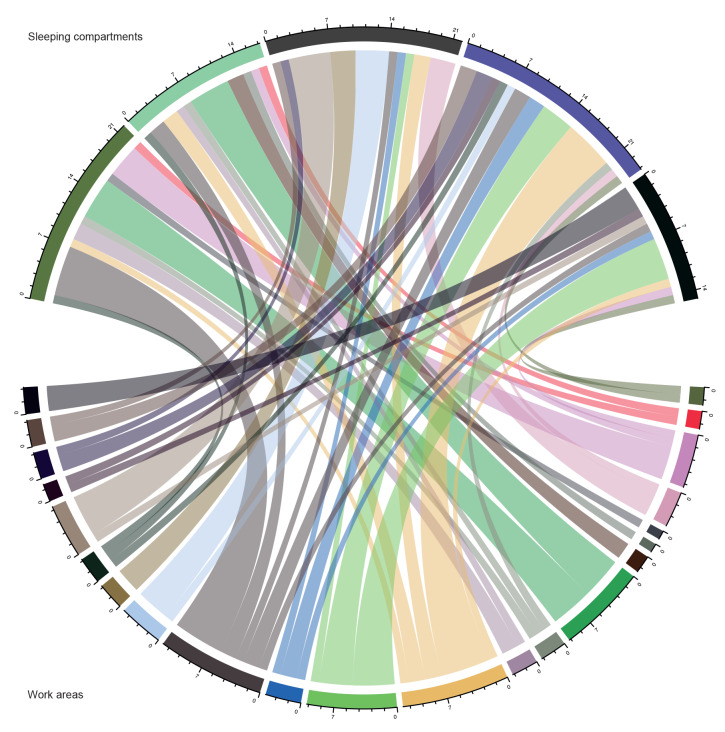
Chord diagram between sleeping compartments and work areas of COVID-19- confirmed cases, Charles de Gaulle aircraft carrier, April 2020 (n = 1,064)

Interviews with the first 30 confirmed cases revealed that two early chains of transmission had started before the stopover in Brest, accounting for the ship’s first eight COVID-19 confirmed cases. The date of symptom onset of the first case was 28 February, leading to only one secondary case among sleeping compartment mates on 11 March (Figure 2). The second index case developed symptoms on 2 March. This case led to five secondary and tertiary cases between 6 and 17 March among individuals working on the flight deck and using a very densely occupied break room. During the 4-day stopover in Brest from 13 to 16 March, 97% of personnel disembarked. Some went to restaurants and bars while others met relatives who came from all over the country. Interviews identified two cases who had symptoms during the stopover, eliciting several additional virus introductions. After the stopover in Brest, SARS-CoV-2 actively circulated on board, and it was difficult to establish true transmission chains. We examined the possible spread of the virus by analysing back-and-forth movements of confirmed cases between sleeping compartments and work areas (Figure 3). Attack rates in work areas were between 56.0% and 78.1%, and in sleeping compartments between 56.9% and 75.0%. Only the flight deck work area and its associated sleeping compartments had a higher attack rate, at 87.5% and 85.0% respectively.

COVID-19: coronavirus disease.

The top half of the chord diagram represents five main sleeping areas. The bottom half represents 16 work areas; each work area was assigned a different colour. The size of the chords spanning across the two halves is proportional to the number of COVID-19-confirmed cases in common between two areas.

### Phylogenetic analyses of subclades

**Figure 4:**
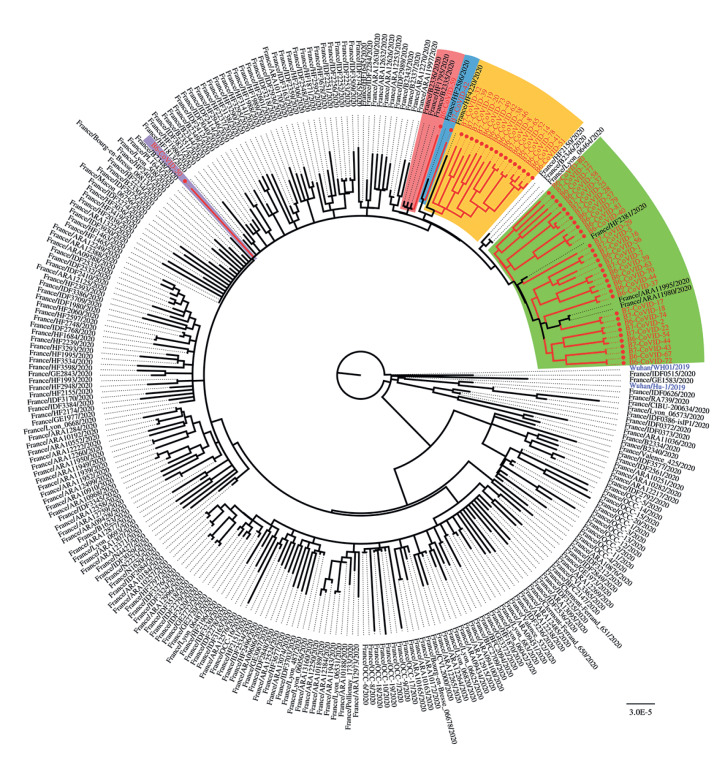
Maximum likelihood tree inferred from sequenced SARS-CoV-2 genomes, Charles de Gaulle aircraft carrier, April 2020 (n = 51), and the French SARS-CoV-2 sequences present in the GISAID database, 3 May 2020 (n = 246)

To further examine viral introduction and spread, we performed phylogenetic and temporal analyses on the 51 strains available among the first 60 SARS-CoV-2-positive samples. Of these 51 strains, five separate groups were identified. Four groups were potentially linked to four separate introduction events (highlighted in blue, green, pink, and yellow in Figure 4; we observed an evolution of the strains with a successive accumulation of mutations. When compared with the known diversity at the time of the outbreak in France, most of the strains clustered with sequences originating from the Brittany, Hauts-de-France, and Auvergne-Rhône-Alpes regions. One strain (highlighted in purple in Figure 4) did not cluster with any other PA-CDG strain. Its positioning confirms that its origin was different than that of all the other 50 genomes analysed, reflecting an additional virus introduction event on the PA-CDG. Among all the samples, we observed no significant correspondence or proximity to isolates originating from Cyprus, Italy, Spain, Portugal, or the United Kingdom (some service members transited through these countries before boarding, see Supplementary Figure S1: Timeline of the main exposure events for SARS-CoV-2 introduction).

SARS-CoV-2: severe acute respiratory syndrome coronavirus 2.

The two SARS-CoV-2 Wuhan strains are coloured in dark blue text. Red circles indicate sequenced genomes from the Charles de Gaulle aircraft carrier. Blue, yellow, pink, purple, and green areas highlight the five different groups of SARS-CoV-2 strains detected on board. The authors acknowledge the originating and submitting laboratories of the sequences from GISAID’s EpiFlu Database used in the phylogenetic analysis.

### Environmental analysis

In an examination of environmental samples, 11 swabs were positive for SARS-CoV-2 among 38 environmental samples collected inside the ship (on shared objects such as a table, handrail, control panel, joystick, toilet) as were several samples from the filters of the air conditioning system. Viral cultures were not performed.

## Discussion

This outbreak in a monitored group of military personnel living in close quarters in a confined environment offered a unique opportunity to describe the natural progression of SARS-CoV-2 epidemic spread and infection characteristics in a young and healthy and predominantly male population. Our case definition, which considers RT-PCR results but also specific clinical signs, was sensitive enough to capture the real proportion of SARS-CoV-2 infected cases. The attack rate was 65%, close to the final 64% seroprevalence measured on the PA-CDG at the end of the 2-week isolation/quarantine period [11].

The outbreak began early in March 2020 among the personnel of the aircraft carrier’s ‘airport’. Case interviews, the epidemic curve, and virus sequencing corroborated the hypothesis of several introductions of the virus on board. Even when at sea, an aircraft carrier is a hub airport that regularly receives aircraft and passengers for the entire CVBG. The first chain of transmission was identified among the flight deck staff, and eventually the highest attack rate was observed in this sub-population. Other introductions occurred during the stopover in Brest 2 weeks later. This stopover represents a critical point in the outbreak. The national lockdown began 2 days later, on 17 March, as SARS-CoV-2 was spreading rapidly in Northern and Eastern France [12]. At that time, access to RT-PCR tests in France was limited. As a rule, ships should avoid stopovers in countries where an epidemic is occurring, and even more so in the case of an emerging pathogen, whose circulation may be underestimated. The results of viral genome sequencing were compatible with the transmission network inferred from the epidemiological data – all the clades identified but one came from France.

The epidemic then spread between sleeping compartments and work areas, moving back and forth, and the measures taken to stop these flows, e.g. remote working, cohorting according to activities, lockdown, caused the spread to slow [12]. This pattern may be the same in the general population between homes and workplaces. Mealtime also played a role in transmission, as the level of proximity in mess halls was associated with probability of infection. During the 15 days following the Brest stopover, preventive measures were forcefully applied, e.g. hand hygiene, decrease in the number of meetings, closing of bars, and particularly, physical distancing [13], which was significantly protective against SARS-CoV-2 infection on the PA-CDG, despite the confined environment and high attack rate, as on the USS-Roosevelt [14]. This also suggests that droplet transmission was predominant in the outbreak. We found viral RNA in the air conditioning system, illustrating the possibility of aerial transmission. Virus exposure through a contaminated environment was also possible, as one out of three environmental samples was positive. However, we cannot conclude on the degree to which aerial or environmental transmission played a role in the spread.

The prevention measures made it possible to contain the speed of virus transmission (1 < R_t_ < 2). As soon as these measures were lifted on 30 March, the outbreak expanded, with R_t_ increasing to almost 3. Eventually, R_t_ declined and the outbreak stopped. Mandatory face mask wearing for the entire crew as well as the systematic isolation of cases occurred well after the epidemic peak, too late to influence its natural course.

Our results suggest rather that once 65% (95% CI: 62–68) of the crew had been infected, that proportion appeared to be large enough to prevent the virus from circulating further. This threshold obtained from a real outbreak was consistent with the level of herd immunity deemed necessary to protect the population from the viral circulation [15]. With the emergence of more-contagious SARS-CoV-2 variants, this threshold would probably have to be raised [16].

We calculated an asymptomatic case rate of 13%, close to that measured on the USS-Roosevelt (19.8%) [17] and in other outbreaks that we have investigated [18,19]. This rate was lower compared with passengers on the Diamond Princess (57.7%), but authors only collected respiratory symptoms [20,21]. More generally, a systematic review carried out by Buitrago et al. highlighted an asymptomatic rate of 20% (95% CI: 17–25) [22]. The low percentage of asymptomatic cases could also have been caused by the very low rate of air renewal on the PA-CDG, as it has been described that the SARS-CoV-2 inoculum could be linked to the clinical profiles of COVID-19 [13].

We had 29 severe cases (2.6%) that required hospitalisation with oxygen, which justified the shortening of the military mission; all recovered. Our findings confirmed that age and BMI were risk factors for increasing COVID-19 intensity [23]. Surprisingly, previous usual active smoking behaviour had lower odds of infection and its severity, although we expected that smoking areas were at risk because of increased close contact. Many studies find that smoking is instead a risk factor for COVID-19 [24-26]. We obtained this result among a young population with no comorbidities, who were not yet suffering from the complications of smoking. According to other studies which found similar results, hypothesised mechanisms of interaction between smoking and COVID-19 were a possible anti-inflammatory effect of nicotine, diminished immune and cytokine responses, or an increase of nitric oxide in the respiratory tract [27,28]. Blood group was not associated with the infection or with its severity.

The ship did not have an isolation ward with a separate ventilation system, so it was not possible to isolate and quarantine the numerous cases and their close contacts on board, once the epidemic was underway. An early warning and countermeasures would have changed the course of the epidemic. The first sign that could have early alerted medical staff was the increase of patients with upper respiratory symptoms and/or anosmia. However, in France, these symptoms were not associated with COVID-19 disease before April 2020. This underlines the important role of epidemiological surveillance [29], in particular syndromic epidemiological surveillance based on pre-diagnosis data collection, for emerging disease detection and management [30]. The deployment of multiplex PCR automates would have made it possible to exclude routine diagnosis as influenza or rhinovirus, leading to the suspicion of an unusual pathogen circulating. In the future, wastewater viral surveillance would also enable early warnings on shipboard viral circulation to be issued [31].

Our study has some limitations. Our findings are from observational retrospective data. Recollection and misrepresentation biases may have led to errors. There were some differences between participants and non-participants. However, selection bias should be limited, as only 11.3% of the services members did not participate to the study. The study group included mainly young males, so that our results cannot be extrapolated to the general population.

## Conclusion

Such an epidemic causes the saturation of care capacities, the decline in the availability of staff and the disorganisation of activities, impacting the operation of the community on board in the end. Communities such as armed forces should protect themselves from such incidents by strengthening adapted prevention and early detection capabilities in order to be better prepared for outbreaks.

## Supplementary Data

665000 Supplement

## References

[1] Migliani R Meynard J-B Milleliri J-M Verret C Rapp C . Histoire de la lutte contre le paludisme dans l’armée française : de l’Algérie à l’Armée d’Orient pendant la Première Guerre mondiale. [History of malaria control in the French armed forces: from Algeria to the Macedonian front during the first World War]. Med Sante Trop. 2014;24(4):349-61. French. 10.1684/mst.2014.0411 25597257

[2] Piarroux R Barrais R Faucher B Haus R Piarroux M Gaudart J Understanding the cholera epidemic, Haiti. Emerg Infect Dis. 2011;17(7):1161-8. 10.3201/eid1707.110059 21762567 PMC3381400

[3] Michel R Demoncheaux JP Créach MA Rapp C Simon F Haus-Cheymol R Prevention of infectious diseases during military deployments: a review of the French armed forces strategy. Travel Med Infect Dis. 2014;12(4):330-40. 10.1016/j.tmaid.2014.07.001 25052855

[4] Bernard Stoecklin S Rolland P Silue Y Mailles A Campese C Simondon A First cases of coronavirus disease 2019 (COVID-19) in France: surveillance, investigations and control measures, January 2020. Euro Surveill. 2020;25(6). 10.2807/1560-7917.ES.2020.25.6.2000094 32070465 PMC7029452

[5] Mizumoto K Kagaya K Zarebski A Chowell G . Estimating the asymptomatic proportion of coronavirus disease 2019 (COVID-19) cases on board the Diamond Princess cruise ship, Yokohama, Japan, 2020. Euro Surveill. 2020;25(10). 10.2807/1560-7917.ES.2020.25.10.2000180 32183930 PMC7078829

[6] Kasper MR Geibe JR Sears CL Riegodedios AJ Luse T Von Thun AM An outbreak of Covid-19 on an aircraft carrier. N Engl J Med. 2020;383(25):2417-26. 10.1056/NEJMoa2019375 33176077 PMC7675688

[7] Guan W-J Ni Z-Y Hu Y Liang WH Ou CQ He JX Clinical characteristics of coronavirus disease 2019 in China. N Engl J Med. 2020;382(18):1708-20. 10.1056/NEJMoa2002032 32109013 PMC7092819

[8] Xie Y Wang Z Liao H Marley G Wu D Tang W . Epidemiologic, clinical, and laboratory findings of the COVID-19 in the current pandemic: systematic review and meta-analysis. BMC Infect Dis. 2020;20(1):640. 10.1186/s12879-020-05371-2 32867706 PMC7457225

[9] Hariyanto TI Rizki NA Kurniawan A . Anosmia/hyposmia is a good predictor of coronavirus disease 2019 (COVID-19) infection: a meta-analysis. Int Arch Otorhinolaryngol. 2021;25(1):e170-4. 10.1055/s-0040-1719120 33552295 PMC7857970

[10] R Core Team. R: A Language and Environment for Statistical Computing. Vienna: R Foundation for Statistical Computing; 2020. Available from: https://www.R-project.org

[11] Bylicki O Paleiron N Janvier F . An outbreak of Covid-19 on an aircraft carrier. N Engl J Med. 2021;384(10):976-7. 10.1056/NEJMc2034424 33567182

[12] Gaudart J Landier J Huiart L Legendre E Lehot L Bendiane MK Factors associated with the spatial heterogeneity of the first wave of COVID-19 in France: a nationwide geo-epidemiological study. Lancet Public Health. 2021;6(4):e222-31. 10.1016/S2468-2667(21)00006-2 33556327 PMC7864788

[13] Bielecki M Züst R Siegrist D Meyerhofer D Crameri GAG Stanga Z Social distancing alters the clinical course of COVID-19 in young adults: a comparative cohort study. Clin Infect Dis. 2021;72(4):598-603. 10.1093/cid/ciaa889 32594121 PMC7337655

[14] Payne DC Smith-Jeffcoat SE Nowak G Chukwuma U Geibe JR Hawkins RJ SARS-CoV-2 Infections and Serologic Responses from a Sample of U.S. Navy Service Members - USS Theodore Roosevelt, April 2020. MMWR Morb Mortal Wkly Rep. 2020;69(23):714-21. 10.15585/mmwr.mm6923e4 32525850 PMC7315794

[15] Randolph HE Barreiro LB . Herd Immunity: Understanding COVID-19. Immunity. 2020;52(5):737-41. 10.1016/j.immuni.2020.04.012 32433946 PMC7236739

[16] Harder T Külper-Schiek W Reda S Treskova-Schwarzbach M Koch J Vygen-Bonnet S Effectiveness of COVID-19 vaccines against SARS-CoV-2 infection with the Delta (B.1.617.2) variant: second interim results of a living systematic review and meta-analysis, 1 January to 25 August 2021. Euro Surveill. 2021;26(41):2100920. 10.2807/1560-7917.ES.2021.26.41.2100920 34651577 PMC8518304

[17] Alvarado GR Pierson BC Teemer ES Gama HJ Cole RD Jang SS . Symptom characterization and outcomes of sailors in isolation after a COVID-19 outbreak on a US aircraft carrier. JAMA Netw Open. 2020;3(10):e2020981. 10.1001/jamanetworkopen.2020.20981 33001200 PMC7530629

[18] de Laval F Grosset-Janin A Delon F Allonneau A Tong C Letois F Lessons learned from the investigation of a COVID-19 cluster in Creil, France: effectiveness of targeting symptomatic cases and conducting contact tracing around them. BMC Infect Dis. 2021;21(1):457. 10.1186/s12879-021-06166-9 34011278 PMC8133048

[19] Durand GA de Laval F de Bonet d’Oléon A Le Flem FX Morin Y Badaut C COVID-19 outbreak among French firefighters, Marseille, France, 2020. Euro Surveill. 2021;26(41):2001676. 10.2807/1560-7917.ES.2021.26.41.2001676 34651571 PMC8518307

[20] Expert Taskforce for the COVID-19 Cruise Ship Outbreak . Epidemiology of COVID-19 outbreak on cruise ship quarantined at Yokohama, Japan, February 2020. Emerg Infect Dis. 2020;26(11):2591-7. 10.3201/eid2611.201165 32822290 PMC7588545

[21] Yamagishi T Kamiya H Kakimoto K Suzuki M Wakita T . Descriptive study of COVID-19 outbreak among passengers and crew on Diamond Princess cruise ship, Yokohama Port, Japan, 20 January to 9 February 2020. Euro Surveill. 2020;25(23):2000272. 10.2807/1560-7917.ES.2020.25.23.2000272 32553062 PMC7403638

[22] Buitrago-Garcia D Egli-Gany D Counotte MJ Hossmann S Imeri H Ipekci AM Occurrence and transmission potential of asymptomatic and presymptomatic SARS-CoV-2 infections: A living systematic review and meta-analysis. PLoS Med. 2020;17(9):e1003346. 10.1371/journal.pmed.1003346 32960881 PMC7508369

[23] Wolff D Nee S Hickey NS Marschollek M . Risk factors for Covid-19 severity and fatality: a structured literature review. Infection. 2021;49(1):15-28. 10.1007/s15010-020-01509-1 32860214 PMC7453858

[24] Patanavanich R Glantz SA . Smoking is associated with COVID-19 progression: a meta-analysis. Nicotine Tob Res. 2020;22(9):1653-6. 10.1093/ntr/ntaa082 32399563 PMC7239135

[25] Prinelli F Bianchi F Drago G Ruggieri S Sojic A Jesuthasan N Association between smoking and SARS-CoV-2 Infection: cross-sectional study of the EPICOVID19 internet-based survey. JMIR Public Health Surveill. 2021;7(4):e27091. 10.2196/27091 33668011 PMC8081027

[26] Qureshi AI Baskett WI Huang W Lobanova I Hasan Naqvi S Shyu C-R . Reinfection with severe acute respiratory syndrome coronavirus 2 (SARS-CoV-2) in patients undergoing serial laboratory testing. Clin Infect Dis. 2022;74(2):294-300. 10.1093/cid/ciab345 33895814 PMC8135382

[27] Xie J Zhong R Wang W Chen O Zou Y . COVID-19 and smoking: what evidence needs our attention? Front Physiol. 2021;12:603850. 10.3389/fphys.2021.603850 33815131 PMC8012895

[28] Usman MS Siddiqi TJ Khan MS Patel UK Shahid I Ahmed J Is there a smoker’s paradox in COVID-19? BMJ Evid Based Med. 2021;26(6):279-84. 10.1136/bmjebm-2020-111492 32788164

[29] Morgan OW Aguilera X Ammon A Amuasi J Fall IS Frieden T Disease surveillance for the COVID-19 era: time for bold changes. Lancet. 2021;397(10292):2317-9. 10.1016/S0140-6736(21)01096-5 34000258 PMC8121493

[30] Caserio-Schönemann C Meynard JB . Ten years experience of syndromic surveillance for civil and military public health, France, 2004-2014. Euro Surveill. 2015;20(19):35-8. 10.2807/1560-7917.ES2015.20.19.21126 25990360

[31] Ahmed W Bertsch PM Angel N Bibby K Bivins A Dierens L Detection of SARS-CoV-2 RNA in commercial passenger aircraft and cruise ship wastewater: a surveillance tool for assessing the presence of COVID-19 infected travellers. J Travel Med. 2020;27(5):taaa116. 10.1093/jtm/taaa116 32662867 PMC7454825

[32] Haas EJ Angulo FJ McLaughlin JM Anis E Singer SR Khan F Impact and effectiveness of mRNA BNT162b2 vaccine against SARS-CoV-2 infections and COVID-19 cases, hospitalisations, and deaths following a nationwide vaccination campaign in Israel: an observational study using national surveillance data. Lancet. 2021;397(10287):1819-29. 10.1016/S0140-6736(21)00947-8 33964222 PMC8099315

