# Supplementary material for: Investigation of a COVID-19 outbreak on the Charles de Gaulle aircraft carrier, March to April 2020: a retrospective cohort study
Source: Euro Surveill. 2022 May 26;27(21):2100612. doi: 10.2807/1560-7917.ES.2022.27.21.2100612 (PMC9137271; doi:10.2807/1560-7917.ES.2022.27.21.2100612)
Supplement: Supplement [file 21-00612_DELAVAL_Supplement.pdf]

SUPPLEMENTARY MATERIAL

INVESTIGATION OF A COVID-19 OUTBREAK ON THE CHARLES DE GAULLE AIRCRAFT CARRIER, A RETROSPECTIVE COHORT STUDY, MARCH-APRIL 2020

This supplementary material is hosted by *Eurosurveillance* as supporting information alongside the article "INVESTIGATION OF A COVID-19 OUTBREAK ON THE CHARLES DE GAULLE AIRCRAFT CARRIER, A RETROSPECTIVE COHORT STUDY, MARCH-APRIL 2020", on behalf of the authors, who remain responsible for the accuracy and appropriateness of the content. The same standards for ethics, copyright, attributions and permissions as for the article apply. Supplements are not edited by *Eurosurveillance* and the journal is not responsible for the maintenance of any links or email addresses provided therein.

Additional method

Study design

Timeline of the main exposure events for SARS-CoV-2 introduction among the crew of the French aircraft carrier Porte-avions Charles de Gaulle, countermeasures put in place, and detection of COVID-19 cases – 19 February – 14 April, 2020 (Figure S1).

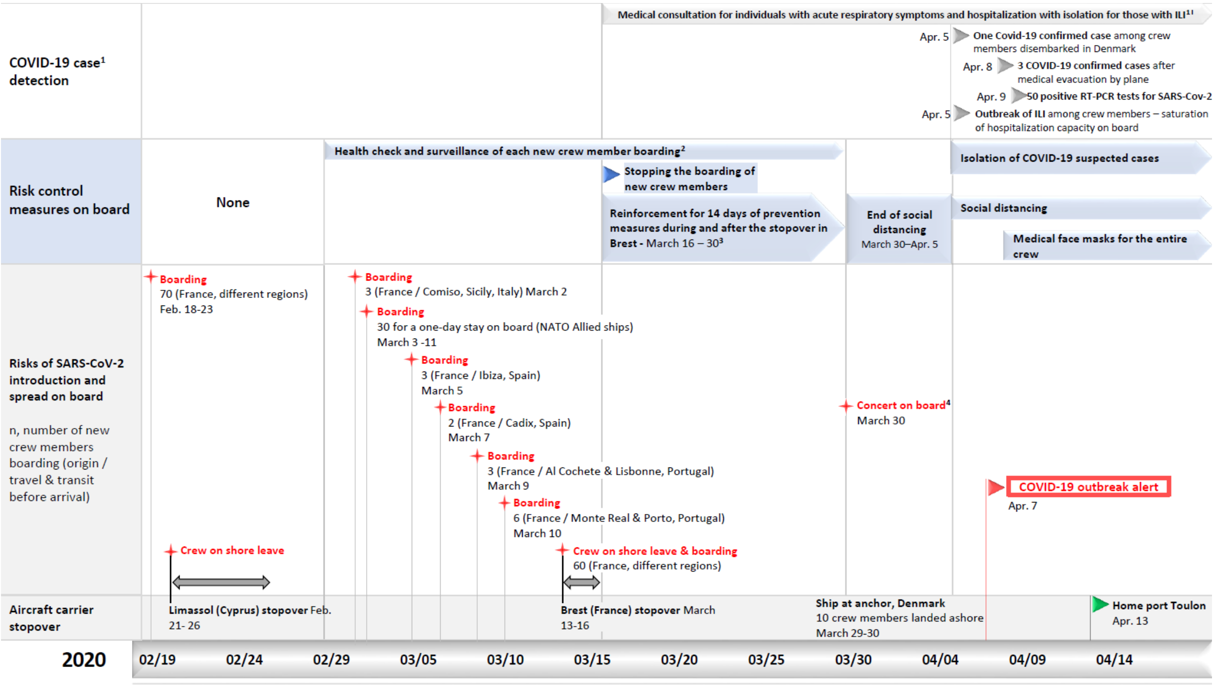

Figure S1: Timeline of the main exposure events for SARS-CoV-2 introduction among the crew of the French aircraft carrier *Porte-avions Charles de Gaulle*, countermeasures put in place, and detection of COVID-19 cases – 19 February – 14 April, 2020.

[1] No biological SARS-CoV-2 diagnosis capability was available on board at this stage of the COVID-19 pandemic. Influenza-like-illness (ILI) was the main syndrome used by the medical staff on board to detect possible COVID-19 cases. Reverse-transcriptase polymerase chain reaction (RT-PCR) tests were performed in France using nasopharyngeal swabs collected on board.

[2] Systematically performed for each new crew member upon arrival on the aircraft carrier (flown in by plane or who boarded during a stopover): evaluation of SARS-CoV-2 exposure in the 14 previous days, medical examination if they presented with respiratory symptoms, isolation in a single room in the ship's medical facility, and face mask for individuals with fever and/or cough until 48 hours after the disappearance of those symptoms.

[3] List of measures implemented during and after the stopover in Brest:
- No family members allowed on board,
- Interviewing of all crew members to identify possible exposure to SARS-CoV-2 during the stopover,
- Medical examination and twice daily temperature checks for 350 crew members who may have been exposed to SARS-CoV-2 during the stopover, and isolation as described in [1],
- Social distancing when possible, including bans on gatherings, limiting briefings, restricting the number of people in gyms, bars, and canteens,

Reinforced hygiene with hand washing and twice daily cleaning of door handles, railings, food service areas, phones, and surfaces.

Viral genome sequencing

Viral genome sequencing was performed on the first 60 positive samples taken on April 8 to contribute to molecular epidemiological studies. The construction of the libraries and the sequencing were carried out according to an in-house protocol adapted based on the ARTIC protocol described by Tyson et al. (1). Sequencing was performed on an Illumina MiSeq with Reagent kit V3 (600-cycle) following the supplier's recommendations. Sequences were obtained by using a custom analysis pipeline, inspired by the nCoV2019 novel coronavirus bioinformatics protocol provided by the Artic network (artic.network/ncov-2019/ncov2019-boinformatics-sop.html). Genomes were then analyzed by comparing the nucleotide sequence of 29.9 kb to that of the Wuhan reference strain (MN908947.3). The pipeline used Trimmomatic (trimming – v0,39) (2), cutPrimers (primer cleaning -v20) (3), PEAR (reads merging – v0,9,6) (4), samtools (v1,9), and BRESEQ (read mapping and variant calling – v0,35,1) (5).
Phylogenetic reconstruction was performed using the Nextstrain analysis pipeline (6). In short, the sequences were aligned using MAFFT v7.463 (7). A maximum likelihood phylogeny was estimated using IQ-TREE multicore version 2.1.1 COVID-edition v1.6.12 (8) with a GTR substitution model, followed by TreeTime v0.7.4 (9) to obtain a time-resolved tree. Sequence Wuhan/WH01/2019 was used as an outgroup to root the tree, which was visualized using FigTree v1.4.4 (http://tree.bio.ed.ac.uk/software/figtree) and edited manually. The genomes available on GISAID at the time of the outbreak were downloaded for this analysis (https://www.gisaid.org/).

Study of the instantaneous reproduction rate

We studied the outbreak dynamics based on the epidemic curves and the instantaneous reproduction rate Rt (10). This Rt value made it possible to measure the instantaneous speed of the epidemic's spread (11) and to detect any significant changes, using a structural change analysis of the underlying linear models (12). We used a calculation time window of Rt equal

to the serial interval published by Bi et al. (13). A sensitivity analysis showed that if this serial interval was made to vary according to the different published values, the Rt curve was only slightly influenced in its amplitude, and not influenced at all over time. We tested several hypotheses to explain the introduction of the virus: we present the most probable hypothesis of a single index case at the start of the curve, plus several introductions during the Brest stopover.

Additional results

Viral genome sequencing was performed on the first 60 positive samples taken from the service members on 8 April to contribute to molecular epidemiological studies; 51 strains were available among the 60 SARS-CoV-2-positive samples. Viral sequences are available online in the GISAID database (https://www.gisaid.org). The anonymous individual identifiers, GISAID accession numbers, and other data can be found in the Supplementary Table S1.

Table S1: Anonymous individual identifiers, and GISAID accession numbers of viral genome sequencings, Charles de Gaulle aircraft carrier, April 2020 (n = 51)

strain	virus	gisaid_epi_isl	date	region	country	division	region_exposure	country_exposure	division_exposure	segment	length	host	pangolin_lineage	GISAID_clade	originating_lab	submitting_lab	url	date_submitted	purpose_of_sequencing
hCoV-19/France/BRE-B6-37/2020	betacoronavirus	EPI_ISL_1797550	2020-04-08	Europe	France	Bretagne	Europe	France	Bretagne	genome	29868	Human	B.1	GH	Medical Center, Aircraft Carrier	IRBA, 2MI	https://www.gisaid.org/	2021-04-29	outbreak investigation
hCoV-19/France/BRE-B6-38/2020	betacoronavirus	EPI_ISL_1797551	2020-04-08	Europe	France	Bretagne	Europe	France	Bretagne	genome	29849	Human	B.1	GH	Medical Center, Aircraft Carrier	IRBA, 2MI	https://www.gisaid.org/	2021-04-29	outbreak investigation
hCoV-19/France/BRE-B6-39/2020	betacoronavirus	EPI_ISL_1797552	2020-04-08	Europe	France	Bretagne	Europe	France	Bretagne	genome	29862	Human	B.1	GH	Medical Center, Aircraft Carrier	IRBA, 2MI	https://www.gisaid.org/	2021-04-29	outbreak investigation
hCoV-19/France/BRE-B6-40/2020	betacoronavirus	EPI_ISL_1797553	2020-04-08	Europe	France	Bretagne	Europe	France	Bretagne	genome	29849	Human	B.1	GH	Medical Center, Aircraft Carrier	IRBA, 2MI	https://www.gisaid.org/	2021-04-29	outbreak investigation
hCoV-19/France/BRE-B6-41/2020	betacoronavirus	EPI_ISL_1797554	2020-04-08	Europe	France	Bretagne	Europe	France	Bretagne	genome	29893	Human	B.1	GH	Medical Center, Aircraft Carrier	IRBA, 2MI	https://www.gisaid.org/	2021-04-29	outbreak investigation
hCoV-19/France/BRE-B6-44/2020	betacoronavirus	EPI_ISL_1797556	2020-04-08	Europe	France	Bretagne	Europe	France	Bretagne	genome	29891	Human	B.1	GH	Medical Center, Aircraft Carrier	IRBA, 2MI	https://www.gisaid.org/	2021-04-29	outbreak investigation
hCoV-19/France/BRE-B6-46/2020	betacoronavirus	EPI_ISL_1797557	2020-04-08	Europe	France	Bretagne	Europe	France	Bretagne	genome	29849	Human	B.1	GH	Medical Center, Aircraft Carrier	IRBA, 2MI	https://www.gisaid.org/	2021-04-29	outbreak investigation
hCoV-19/France/BRE-B6-49/2020	betacoronavirus	EPI_ISL_1797558	2020-04-08	Europe	France	Bretagne	Europe	France	Bretagne	genome	29803	Human	B.1	GH	Medical Center, Aircraft Carrier	IRBA, 2MI	https://www.gisaid.org/	2021-04-29	outbreak investigation
hCoV-19/France/BRE-B6-50/2020	betacoronavirus	EPI_ISL_1797559	2020-04-08	Europe	France	Bretagne	Europe	France	Bretagne	genome	29867	Human	B.1	GH	Medical Center, Aircraft Carrier	IRBA, 2MI	https://www.gisaid.org/	2021-04-29	outbreak investigation
hCoV-19/France/BRE-B6-51/2020	betacoronavirus	EPI_ISL_1797560	2020-04-08	Europe	France	Bretagne	Europe	France	Bretagne	genome	29867	Human	B.1	GH	Medical Center, Aircraft Carrier	IRBA, 2MI	https://www.gisaid.org/	2021-04-29	outbreak investigation
hCoV-19/France/BRE-B6-52/2020	betacoronavirus	EPI_ISL_1797561	2020-04-08	Europe	France	Bretagne	Europe	France	Bretagne	genome	29872	Human	B.1	GH	Medical Center, Aircraft Carrier	IRBA, 2MI	https://www.gisaid.org/	2021-04-29	outbreak investigation
hCoV-19/France/BRE-B6-54/2020	betacoronavirus	EPI_ISL_1797562	2020-04-08	Europe	France	Bretagne	Europe	France	Bretagne	genome	29849	Human	B.1	GH	Medical Center, Aircraft Carrier	IRBA, 2MI	https://www.gisaid.org/	2021-04-29	outbreak investigation
hCoV-19/France/BRE-B6-56/2020	betacoronavirus	EPI_ISL_1797563	2020-04-08	Europe	France	Bretagne	Europe	France	Bretagne	genome	29885	Human	B.1	GH	Medical Center, Aircraft Carrier	IRBA, 2MI	https://www.gisaid.org/	2021-04-29	outbreak investigation
hCoV-19/France/BRE-B6-57/2020	betacoronavirus	EPI_ISL_1797564	2020-04-08	Europe	France	Bretagne	Europe	France	Bretagne	genome	29800	Human	B.1	GH	Medical Center, Aircraft Carrier	IRBA, 2MI	https://www.gisaid.org/	2021-04-29	outbreak investigation
hCoV-19/France/BRE-B6-58/2020	betacoronavirus	EPI_ISL_1797565	2020-04-08	Europe	France	Bretagne	Europe	France	Bretagne	genome	29886	Human	B.1	GH	Medical Center, Aircraft Carrier	IRBA, 2MI	https://www.gisaid.org/	2021-04-29	outbreak investigation
hCoV-19/France/BRE-B6-59/2020	betacoronavirus	EPI_ISL_1797566	2020-04-08	Europe	France	Bretagne	Europe	France	Bretagne	genome	29876	Human	B.1	GH	Medical Center, Aircraft Carrier	IRBA, 2MI	https://www.gisaid.org/	2021-04-29	outbreak investigation
hCoV-19/France/BRE-B6-62/2020	betacoronavirus	EPI_ISL_1797567	2020-04-08	Europe	France	Bretagne	Europe	France	Bretagne	genome	29875	Human	B.1	GH	Medical Center, Aircraft Carrier	IRBA, 2MI	https://www.gisaid.org/	2021-04-29	outbreak investigation
hCoV-19/France/BRE-B6-63/2020	betacoronavirus	EPI_ISL_1797568	2020-04-08	Europe	France	Bretagne	Europe	France	Bretagne	genome	29893	Human	B.1	GH	Medical Center, Aircraft Carrier	IRBA, 2MI	https://www.gisaid.org/	2021-04-29	outbreak investigation
hCoV-19/France/BRE-B6-65/2020	betacoronavirus	EPI_ISL_1797569	2020-04-08	Europe	France	Bretagne	Europe	France	Bretagne	genome	29849	Human	B.1	GH	Medical Center, Aircraft Carrier	IRBA, 2MI	https://www.gisaid.org/	2021-04-29	outbreak investigation
hCoV-19/France/BRE-B6-67/2020	betacoronavirus	EPI_ISL_1797570	2020-04-08	Europe	France	Bretagne	Europe	France	Bretagne	genome	29873	Human	B.1	GH	Medical Center, Aircraft Carrier	IRBA, 2MI	https://www.gisaid.org/	2021-04-29	outbreak investigation
hCoV-19/France/BRE-B6-70/2020	betacoronavirus	EPI_ISL_1797571	2020-04-08	Europe	France	Bretagne	Europe	France	Bretagne	genome	29871	Human	B.1	GH	Medical Center, Aircraft Carrier	IRBA, 2MI	https://www.gisaid.org/	2021-04-29	outbreak investigation
hCoV-19/France/BRE-B6-72/2020	betacoronavirus	EPI_ISL_1797572	2020-04-08	Europe	France	Bretagne	Europe	France	Bretagne	genome	29866	Human	B.1	GH	Medical Center, Aircraft Carrier	IRBA, 2MI	https://www.gisaid.org/	2021-04-29	outbreak investigation
hCoV-19/France/BRE-B6-73/2020	betacoronavirus	EPI_ISL_1797573	2020-04-08	Europe	France	Bretagne	Europe	France	Bretagne	genome	29849	Human	B.1	GH	Medical Center, Aircraft Carrier	IRBA, 2MI	https://www.gisaid.org/	2021-04-29	outbreak investigation
hCoV-19/France/BRE-B6-74/2020	betacoronavirus	EPI_ISL_1797574	2020-04-08	Europe	France	Bretagne	Europe	France	Bretagne	genome	29849	Human	B.1	GH	Medical Center, Aircraft Carrier	IRBA, 2MI	https://www.gisaid.org/	2021-04-29	outbreak investigation
hCoV-19/France/BRE-B6-77/2020	betacoronavirus	EPI_ISL_1797575	2020-04-08	Europe	France	Bretagne	Europe	France	Bretagne	genome	29782	Human	B.1	GH	Medical Center, Aircraft Carrier	IRBA, 2MI	https://www.gisaid.org/	2021-04-29	outbreak investigation
hCoV-19/France/BRE-B6-78/2020	betacoronavirus	EPI_ISL_1797576	2020-04-08	Europe	France	Bretagne	Europe	France	Bretagne	genome	29795	Human	B.1	GH	Medical Center, Aircraft Carrier	IRBA, 2MI	https://www.gisaid.org/	2021-04-29	outbreak investigation
hCoV-19/France/BRE-B6-79/2020	betacoronavirus	EPI_ISL_1797577	2020-04-08	Europe	France	Bretagne	Europe	France	Bretagne	genome	29865	Human	B.1	GH	Medical Center, Aircraft Carrier	IRBA, 2MI	https://www.gisaid.org/	2021-04-29	outbreak investigation
hCoV-19/France/BRE-B6-80/2020	betacoronavirus	EPI_ISL_1797578	2020-04-08	Europe	France	Bretagne	Europe	France	Bretagne	genome	29868	Human	B.1	GH	Medical Center, Aircraft Carrier	IRBA, 2MI	https://www.gisaid.org/	2021-04-29	outbreak investigation
hCoV-19/France/BRE-B6-81/2020	betacoronavirus	EPI_ISL_1797579	2020-04-08	Europe	France	Bretagne	Europe	France	Bretagne	genome	29894	Human	B.1	GH	Medical Center, Aircraft Carrier	IRBA, 2MI	https://www.gisaid.org/	2021-04-29	outbreak investigation
hCoV-19/France/BRE-B7-01/2020	betacoronavirus	EPI_ISL_1797580	2020-04-08	Europe	France	Bretagne	Europe	France	Bretagne	genome	29849	Human	B.1	GH	Medical Center, Aircraft Carrier	IRBA, 2MI	https://www.gisaid.org/	2021-04-29	outbreak investigation
hCoV-19/France/BRE-B7-02/2020	betacoronavirus	EPI_ISL_1797588	2020-04-08	Europe	France	Bretagne	Europe	France	Bretagne	genome	29862	Human	B.1	GH	Medical Center, Aircraft Carrier	IRBA, 2MI	https://www.gisaid.org/	2021-04-29	outbreak investigation
hCoV-19/France/BRE-B7-05/2020	betacoronavirus	EPI_ISL_1797600	2020-04-08	Europe	France	Bretagne	Europe	France	Bretagne	genome	29866	Human	B.1	GH	Medical Center, Aircraft Carrier	IRBA, 2MI	https://www.gisaid.org/	2021-04-29	outbreak investigation
hCoV-19/France/BRE-B7-07/2020	betacoronavirus	EPI_ISL_1797601	2020-04-08	Europe	France	Bretagne	Europe	France	Bretagne	genome	29849	Human	B.1	GH	Medical Center, Aircraft Carrier	IRBA, 2MI	https://www.gisaid.org/	2021-04-29	outbreak investigation
hCoV-19/France/BRE-B7-08/2020	betacoronavirus	EPI_ISL_1797602	2020-04-08	Europe	France	Bretagne	Europe	France	Bretagne	genome	29849	Human	B.1	GH	Medical Center, Aircraft Carrier	IRBA, 2MI	https://www.gisaid.org/	2021-04-29	outbreak investigation
hCoV-19/France/BRE-B7-12/2020	betacoronavirus	EPI_ISL_1797582	2020-04-08	Europe	France	Bretagne	Europe	France	Bretagne	genome	29868	Human	B.1	GH	Medical Center, Aircraft Carrier	IRBA, 2MI	https://www.gisaid.org/	2021-04-29	outbreak investigation
hCoV-19/France/BRE-B7-14/2020	betacoronavirus	EPI_ISL_1797583	2020-04-08	Europe	France	Bretagne	Europe	France	Bretagne	genome	29782	Human	B.1	GH	Medical Center, Aircraft Carrier	IRBA, 2MI	https://www.gisaid.org/	2021-04-29	outbreak investigation
hCoV-19/France/BRE-B7-16/2020	betacoronavirus	EPI_ISL_1797584	2020-04-08	Europe	France	Bretagne	Europe	France	Bretagne	genome	29867	Human	B.1	GH	Medical Center, Aircraft Carrier	IRBA, 2MI	https://www.gisaid.org/	2021-04-29	outbreak investigation
hCoV-19/France/BRE-B7-17/2020	betacoronavirus	EPI_ISL_1797585	2020-04-08	Europe	France	Bretagne	Europe	France	Bretagne	genome	29901	Human	B.1	GH	Medical Center, Aircraft Carrier	IRBA, 2MI	https://www.gisaid.org/	2021-04-29	outbreak investigation
hCoV-19/France/BRE-B7-18/2020	betacoronavirus	EPI_ISL_1797586	2020-04-08	Europe	France	Bretagne	Europe	France	Bretagne	genome	29867	Human	B.1	GH	Medical Center, Aircraft Carrier	IRBA, 2MI	https://www.gisaid.org/	2021-04-29	outbreak investigation
hCoV-19/France/BRE-B7-19/2020	betacoronavirus	EPI_ISL_1797587	2020-04-08	Europe	France	Bretagne	Europe	France	Bretagne	genome	29867	Human	B.1	GH	Medical Center, Aircraft Carrier	IRBA, 2MI	https://www.gisaid.org/	2021-04-29	outbreak investigation
hCoV-19/France/BRE-B7-20/2020	betacoronavirus	EPI_ISL_1797589	2020-04-08	Europe	France	Bretagne	Europe	France	Bretagne	genome	29860	Human	B.1	GH	Medical Center, Aircraft Carrier	IRBA, 2MI	https://www.gisaid.org/	2021-04-29	outbreak investigation
hCoV-19/France/BRE-B7-22/2020	betacoronavirus	EPI_ISL_1797590	2020-04-08	Europe	France	Bretagne	Europe	France	Bretagne	genome	29892	Human	B.1	GH	Medical Center, Aircraft Carrier	IRBA, 2MI	https://www.gisaid.org/	2021-04-29	outbreak investigation
hCoV-19/France/BRE-B7-25/2020	betacoronavirus	EPI_ISL_1797591	2020-04-08	Europe	France	Bretagne	Europe	France	Bretagne	genome	29862	Human	B.1	GH	Medical Center, Aircraft Carrier	IRBA, 2MI	https://www.gisaid.org/	2021-04-29	outbreak investigation
hCoV-19/France/BRE-B7-28/2020	betacoronavirus	EPI_ISL_1797592	2020-04-08	Europe	France	Bretagne	Europe	France	Bretagne	genome	29872	Human	B.1	GH	Medical Center, Aircraft Carrier	IRBA, 2MI	https://www.gisaid.org/	2021-04-29	outbreak investigation
hCoV-19/France/BRE-B7-29/2020	betacoronavirus	EPI_ISL_1797593	2020-04-08	Europe	France	Bretagne	Europe	France	Bretagne	genome	29849	Human	B.1	GH	Medical Center, Aircraft Carrier	IRBA, 2MI	https://www.gisaid.org/	2021-04-29	outbreak investigation
hCoV-19/France/BRE-B7-32/2020	betacoronavirus	EPI_ISL_1797594	2020-04-08	Europe	France	Bretagne	Europe	France	Bretagne	genome	29849	Human	B.1	GH	Medical Center, Aircraft Carrier	IRBA, 2MI	https://www.gisaid.org/	2021-04-29	outbreak investigation
hCoV-19/France/BRE-B7-37/2020	betacoronavirus	EPI_ISL_1797595	2020-04-08	Europe	France	Bretagne	Europe	France	Bretagne	genome	29898	Human	B.1	GH	Medical Center, Aircraft Carrier	IRBA, 2MI	https://www.gisaid.org/	2021-04-29	outbreak investigation
hCoV-19/France/BRE-B7-39/2020	betacoronavirus	EPI_ISL_1797596	2020-04-08	Europe	France	Bretagne	Europe	France	Bretagne	genome	29849	Human	B.1	GH	Medical Center, Aircraft Carrier	IRBA, 2MI	https://www.gisaid.org/	2021-04-29	outbreak investigation
hCoV-19/France/BRE-B7-42/2020	betacoronavirus	EPI_ISL_1797597	2020-04-08	Europe	France	Bretagne	Europe	France	Bretagne	genome	29849	Human	B.1	GH	Medical Center, Aircraft Carrier	IRBA, 2MI	https://www.gisaid.org/	2021-04-29	outbreak investigation
hCoV-19/France/BRE-B7-43/2020	betacoronavirus	EPI_ISL_1797598	2020-04-08	Europe	France	Bretagne	Europe	France	Bretagne	genome	29854	Human	B.1	GH	Medical Center, Aircraft Carrier	IRBA, 2MI	https://www.gisaid.org/	2021-04-29	outbreak investigation
hCoV-19/France/BRE-B7-44/2020	betacoronavirus	EPI_ISL_1797599	2020-04-08	Europe	France	Bretagne	Europe	France	Bretagne	genome	29868	Human	B.1	GH	Medical Center, Aircraft Carrier	IRBA, 2MI	https://www.gisaid.org/	2021-04-29	outbreak investigation

Additional bibliography

1. Tyson JR, James P, Stoddart D, et al. Improvements to the ARTIC multiplex PCR method for SARS-CoV-2 genome sequencing using nanopore. BioRxiv. 2020 Sep 4;2020.09.04.283077. Preprint
2. Bolger AM, Lohse M, Usadel B. Trimmomatic: a flexible trimmer for Illumina sequence data. Bioinforma Oxf Engl. 2014;30(15):2114-20.
3. Kechin A, Boyarskikh U, Kel A, Filipenko M. cutPrimers: A New Tool for Accurate Cutting of Primers from Reads of Targeted Next Generation Sequencing. J Comput Biol J Comput Mol Cell Biol. 2017;24(11):1138-43.
4. Zhang J, Kobert K, Flouri T, Stamatakis A. PEAR: a fast and accurate Illumina Paired-End reAd mergeR. Bioinforma Oxf Engl. 2014;30(5):614-20.
5. Deatherage DE, Barrick JE. Identification of mutations in laboratory-evolved microbes from next-generation sequencing data using breseq. Methods Mol Biol Clifton NJ. 2014;1151:165-88.
6. Hadfield J, Megill C, Bell SM, et al. Nextstrain: real-time tracking of pathogen evolution. Bioinforma Oxf Engl. 2018;34(23):4121-3.
7. Katoh K, Misawa K, Kuma K, Miyata T. MAFFT: a novel method for rapid multiple sequence alignment based on fast Fourier transform. Nucleic Acids Res. 2002;30(14):3059-66.
8. Minh BQ, Schmidt HA, Chernomor O, et al. IQ-TREE 2: New Models and Efficient Methods for Phylogenetic Inference in the Genomic Era. Mol Biol Evol. 2020;37(5):1530-4.
9. Sagulenko P, Puller V, Neher RA. TreeTime: Maximum-likelihood phylodynamic analysis. Virus Evol. 2018;4(1):vex042.
10. Cori A, Ferguson NM, Fraser C, Cauchemez S. A new framework and software to estimate time-varying reproduction numbers during epidemics. Am J Epidemiol. 2013;178(9):1505-12.
11. Fraser C. Estimating individual and household reproduction numbers in an emerging epidemic. PloS One. 2007;2(8):e758.
12. Bai J, Perron P. Computation and analysis of multiple structural change models. J Appl Econom. 2003;18(1):1-22.
13. Bi Q, Wu Y, Mei S, et al. Epidemiology and transmission of COVID-19 in 391 cases and 1286 of their close contacts in Shenzhen, China: a retrospective cohort study. Lancet Infect Dis. 2020;20(8):911-9.
